# From Acute Stress to Long-Term Dysregulation: Changes in Hematological and Hormonal Parameters in the Long-Term Post-Stress Period in a Modified SPS&S Model

**DOI:** 10.3390/biomedicines14020356

**Published:** 2026-02-03

**Authors:** Darya I. Gonchar, Tatiana A. Shmigol, Dmitri N. Lyakhmun, Aleksandra E. Soloveva, Svetlana K. Yankovskaya, Olga V. Krendeleva, Veriko D. Vizgalina, Ekaterina V. Efimova, Aiarpi A. Ezdoglian, Nina M. Kiseleva, Vadim V. Negrebetsky

**Affiliations:** 1 Scientific Testing Center, Institute of Pharmacy and Medicinal Chemistry, Federal State Autonomous Educational Institution of Higher Education «N.I. Pirogov Russian National Research Medical University», Ministry of Health of the Russian Federation, Ostrovityanova, 1-6, 117513 Moscow, Russia; daria.gonchar.1999@mail.ru (D.I.G.); lyahmun@mail.ru (D.N.L.); alexa_sol@mail.ru (A.E.S.); talanova.sk@gmail.com (S.K.Y.); krendeleva.olga0@gmail.com (O.V.K.); verikovizgalina@mail.ru (V.D.V.); ekater118@mail.ru (E.V.E.); kiseleva.67.nina@yandex.ru (N.M.K.); nmr_rsmu@yahoo.com (V.V.N.); 2 Department of Rheumatology and Clinical Immunology, Amsterdam University Medical Center, 1081 HV Amsterdam, The Netherlands; a.ezdoglian@gmail.com

**Keywords:** SPS&S model, PTSD-like behavior, anxiety-like behavior, endocrine dysregulation, hematological parameters

## Abstract

**Objectives**: Existing animal models of post-traumatic stress disorder (PTSD) are often methodologically complex and produce variable outcomes. The aim of this study was to develop a modified PTSD model that accurately recapitulates the clinical progression of the disorder, incorporating both behavioral features and objective physiological parameters. **Methods**: We utilized a modified Single Prolonged Stress with Subsequent Stress (SPS&S) protocol, supplemented by a stress reminder phase (without re-exposure to primary stressors) and an evaluation of stress response extinction. Eighty male Wistar rats were subjected to the stress protocol, followed by comprehensive behavioral, hematological (leukocytes, hemoglobin, and hematocrit), and hormonal (corticosterone; adrenocorticotropic hormone (ACTH)) assessments 4–5 weeks post-stress. **Results**: The model produced a PTSD-like phenotype in 25% of animals, characterized by persistent alterations in the investigated biomarkers. The PTSD group exhibited sustained behavioral impairments (increased anxiety), hematological changes (neutrophilic leukocytosis), and endocrine dysregulation (decreased corticosterone, ACTH, and epinephrine). **Conclusions**: This modified SPS&S model demonstrates validity for studying the long-term consequences of stress, with PTSD markers remaining stable throughout the 28-day observation period.

## 1. Introduction

Modern research increasingly demonstrates that stress is not simply a psychological reaction but a complex systemic process that can lead to persistent disturbance of the body’s homeostasis [1]. In recent decades, particular attention has been paid to studying the mechanisms underlying the transition from an acute stress response to a long-term dysregulation of physiological systems, which is crucial for understanding the pathogenesis of post-traumatic stress disorder (PTSD), depression, and related somatic illnesses [2,3]. The classic Single Prolonged Stress (SPS) model [4], developed for studying PTSD, requires modification to more accurately reproduce the complex pathophysiological changes observed in clinical practice.

A major challenge is the heterogeneity in clinical presentations of PTSD, as only a subset of individuals exposed to trauma develop the full-blown disorder. However, despite its widespread use, the classical SPS model has a significant limitation, noted in several reviews [5,6], namely, the transient nature of behavioral and endocrine changes, which does not fully reflect the chronic nature of PTSD in humans. Furthermore, many studies apply data averaging across entire groups of stressed animals, failing to account for clinically relevant heterogeneity in traumatic stress responses, where the disorder develops only in a subset of individuals [7].

To overcome these limitations, this study utilized a modified Single Prolonged Stress with Subsequent Stress (SPS&S) model [8], which combines elements of acute severe stress with subsequent prolonged exposure to moderate stressors. This approach allows for a more accurate modeling of the dynamics of stress-induced disorders, including changes in hematological parameters and endocrine regulation. Unlike approaches that average data across the entire group, this study focuses on stratifying animals into distinct phenotypic subgroups (vulnerable and resilient to stress) based on a comprehensive behavioral analysis. Particular emphasis is placed on examining the long-term post-stress period (up to 28 days), during which the most persistent pathological changes develop.

Thus, the aim of this study was to develop and comprehensively validate a modified PTSD model that produces a stable and heterogeneous phenotype, while minimizing pronounced comorbid depressive and compulsive manifestations [9].

The relevance of this study is underscored by the need for the following:

1. Identifying reliable biomarkers of stress-induced conditions based on phenotypic stratification;
2. Understanding the mechanisms of transition of adaptive responses into pathological ones, including in vulnerable individuals;
3. Developing new approaches for the prevention and treatment of chronic stress consequences.

## 2. Materials and Methods

### 2.1. Animals

The experiments were carried out on adult male Wistar rats (Federal State Budgetary Scientific Institution “Federal Research Center Institute of Cytology and Genetics of the Siberian Branch of the Russian Academy of Sciences” (ICG SB RAS), Novosibirsk, Russia; 5–6 weeks old, weighing approximately 200 g; n = 100). All rats were obtained as a single batch and were weaned at the standard age (21 days). Individual information regarding litter of origin or specific dam was not recorded, which may be considered a source of uncontrolled variability in the stress response. For such studies, obtaining “ready-made” animals from breeding facilities is a limiting factor. This is due to the fact that the inability to control postnatal exposures in animals reduces the assessment of the severity of both behavioral and hormonally active stress responses in adulthood [10,11]. All animals were obtained as a single shipment batch. Upon arrival, the rats were quarantined and allowed to acclimate to the laboratory environment for two weeks prior to any experimental procedures.

The animals were housed in a conventional vivarium under controlled environmental conditions: temperature 20–24 °C, relative humidity 30–55%, a 12 h light/dark cycle (08:00–20:00 light; 20:00–08:00 dark), and 15 air changes per hour. Food and water were available ad libitum. Rats were housed in standard polycarbonate type IV cages (dimensions 545 × 395 × 200 mm) in groups of 5 animals per cage. During the 12 h sucrose preference test, each animal was temporarily transferred to an individual cage (466 × 314 × 200 mm). Animals were individually marked using a non-phenolic dye applied to the fur. Individual animal markings were refreshed every 3 weeks to ensure reliable identification, especially following water-based procedures. All animals underwent the marking procedure simultaneously.

All experimental procedures were approved by the Animal Care and Use Ethics Committee of the Pirogov Russian National Research Medical University (Protocol No. 04/2025). All animals underwent a handling procedure prior to the start of this study. All experiments were conducted by female researchers, as male researchers often induce a strong physiological stress response in animals [12]. Work with animals was divided among researchers. Stress induction was performed by two researchers who subsequently did not participate in the assessment of animal behavior. Behavioral assessment was conducted by two researchers who were not involved in stressful manipulations or blood sampling, but with whom the animals were familiar. This minimized the stress associated with anticipating the stressful procedure. Blood sampling was performed by one researcher who was not involved in stress induction and did not participate in behavioral assessment. Animal housing and care were the responsibility of one researcher who performed no experimental manipulations according to the study protocol. To minimize variability caused by individual handling styles, all experimenters strictly adhered to standardized protocols.

### 2.2. Experiment Design and Phenotypic Stratification

Rats were assigned to two experimental groups: Group 1—model animals (Model) subjected to stress exposure (n = 80)—and Group 2—intact animals (Control) (n = 20). Since the prenatal and postnatal development of animals in breeding facility conditions represents a clear limitation of this study, group formation was conducted to minimize risks and was based on body weight and baseline activity in the hole-board apparatus (assessed by the number of sectors crossed, number of entries into the center of the apparatus, number of rearings, and head dips into the holes). This study utilized a modified Single Prolonged Stress with Subsequent Stress (SPS&S) model [8], which included stress reminder phases (without repeated exposure to the primary stressors) and an assessment of stress response extinction.

Following the stress procedure, animals in the Model group were stratified into two phenotypic subgroups based on a post hoc analysis performed by a researcher blinded to subsequent biochemical assays. The stratification employed a combination of behavioral and endocrine criteria assessed during weeks 1–3, designed to capture the hallmark dynamics of PTSD-like development:

Behavioral Avoidance. Time spent in the closed arms of the Elevated Plus Maze (EPM) at the end of week 1 had to exceed the mean value of the Control group.

Acute Stress Hormonal Response. Plasma corticosterone level measured 24 h after the initial SPS&S procedure (week 1) had to be higher than the mean of the Control group.

Delayed Hormonal Dysregulation. Plasma corticosterone level at week 3 had to be lower than the mean of the Control group.

**Scheme 1:**
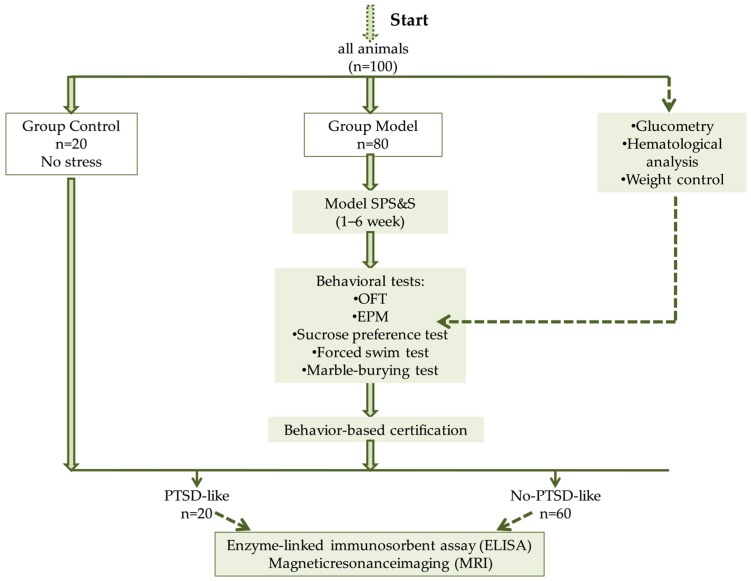
Diagram of the experimental design.

Rats meeting all three criteria were classified as having a PTSD-like phenotype (PTSD-like, n = 20). The remaining animals from the Model group, which did not meet the full set of criteria, were classified as the No-PTSD-like subgroup (n = 60) (Scheme 1).

### 2.3. Stress Procedures

**Scheme 2:**
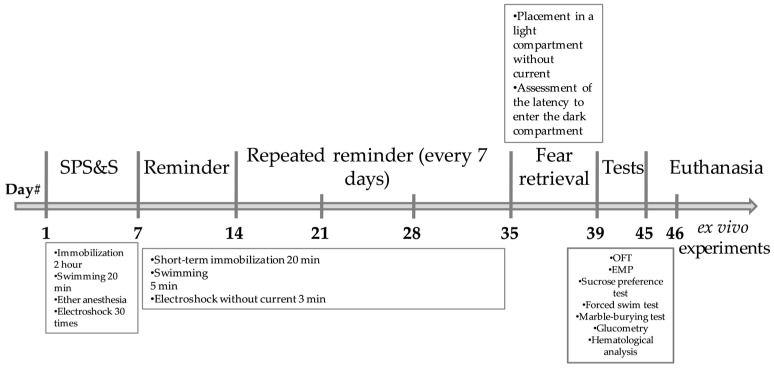
Line diagram of the experimental design.

All stress manipulations for the Model group were performed in the morning from 8:00 to 11:00. To minimize stress exposure to the control animals, all stress procedures were conducted in a separate, isolated room that prevented the transmission of odors and noise (Scheme 2).

#### 2.3.1. Acute Prolonged Stress Phase (On Week 1, Day 1)

Model group animals (n = 80) were subjected to a sequence of stressors:

1. Immobilization: Rats were placed in restrainers that almost completely immobilized them for 2 h.
2. Forced Swimming: Immediately after immobilization, animals were placed in cylinders for 20 min.
3. Ether Anesthesia: After a 15 min recovery period, rats were exposed to diethyl ether vapor until the loss of the pain reflex.
4. Electric Shock: After recovering from anesthesia, animals were placed in the dark compartment of a conditioned avoidance chamber (580 × 487 × 330 mm, Neurobotics LLC, Moscow, Russia). Following a 2 min habituation period, they received 30 electric foot shocks (1.5 mA, 1 s duration, and random inter-shock interval of 30–60 s). The animals were returned to their home cages 60 s after the final shock.
5. For the next 7 consecutive days, the rats were left undisturbed.

#### 2.3.2. Stress Reminder Phase (On Weeks 2–5; Days 7, 14, 21, and 28)

1. Immobilization. Rats were placed in restraints that completely/almost completely immobilized the animals for 20 min
2. Forced swimming. Immediately after immobilization, animals were placed in cylinders, in which the animals swam for 5 min
3. Electric shock. After recovery from anesthesia, animals were placed in the dark compartment of a conditioned avoidance chamber (580 × 487 × 330 mm, Neurobotics LLC, Moscow, Russia) for 3 min without current application.

The procedure was repeated 4 times at 7-day intervals.

#### 2.3.3. Extinction Phase (On Week 6, Day 35–38)

During week 6, stress extinction was assessed over 4 days. In this phase, animals were placed in a box (light compartment) without shock delivery, from which they were moved to the dark zone where they remembered being shocked. The latency to enter the dark compartment was assessed on day 4.

### 2.4. Physiological and Biochemical Assessments

The following assessments were performed weekly throughout this study: body weight measurement, glucose monitoring, complete blood count (CBC), and the sucrose preference test.

#### 2.4.1. Body Weight and Sucrose Preference Test (SPT)

Animals were weighed weekly (AND GP-20K scales, A&D Company Ltd., Tokyo, Japan). Analysis of body weight changes was performed using the “Body weight gain” parameter. This parameter was calculated relative to body weight before the start of this study. The calculation was performed using the following formula:

$$Body\;weight\;gain,\%=\;\frac{\left(m_{n}-m_{0}\right)}{m_{0}}\times 100\%$$

where

*m*_0_—animal body weight at the beginning of the study, g;

*m*_*n*_—animal body weight at a specific time point, g.

The sucrose preference test, used to assess anhedonia, was conducted as described above [13]. Prior to testing, the animals were trained for two days: on the first day, two bottles with a 1% sucrose solution were placed in each cage for 24 h; on the second day, one bottle was replaced with regular water for 24 h. Before the actual testing, animals were water-deprived for 6 h (not applied on training days). During the test, two pre-weighed bottles were provided for 12 h—one with a 1% sucrose solution and the other with regular water. Sucrose preference was calculated as the ratio of sucrose consumed to the total fluid intake expressed as a percentage.

#### 2.4.2. Hematological Parameters and Glucose Level in Peripheral Blood

Weekly blood sampling was performed from the sublingual vein. The detailed Standard Operating Procedure for sublingual vein blood sampling is provided in the Supplementary Material File S1. Blood glucose levels were measured immediately using a Contour TS glucometer (Ascensia Diabetes Care Holdings AG, Basel, Switzerland). Hematological analysis was conducted within 2–3 h post-sampling using an Abacus Junior Vet 5 analyzer (Diatron, Vienna, Austria).

#### 2.4.3. Enzyme-Linked Immunosorbent Assay (ELISA)

Blood samples were allowed to clot at room temperature and then centrifuged at 3000 rpm for 5 min (rotor bucket LL062 18293, centrifuge 5804R, Eppendorf, Hamburg, Germany). The serum was aliquoted and stored at −80 °C until analysis. Following euthanasia, brains were collected, and hippocampi were rapidly dissected on ice, frozen in liquid nitrogen, and stored at −80 °C. Serum levels of corticosterone and adrenocorticotropic hormone (ACTH), as well as adrenaline levels in hippocampal homogenates, were measured using commercial ELISA kits (LK Biotechnology Co., Ltd., Hangzhou, China) according to the manufacturer’s protocols.

#### 2.4.4. Magnetic Resonance Imaging (MRI)

A single in vivo magnetic resonance imaging (MRI) session was conducted on a Bruker BioSpin ClinScan system (Bruker Biospin MRI GmbH, Reinstetten, Germany) to quantify hippocampal volume in animals under general isoflurane anesthesia. Three researchers, blinded to the experimental groups, independently analyzed the MRI data. The volume of the hippocampus was determined on consecutive slices using Radiant DICOM Viewer software (https://www.radiantviewer.com, version: 2023.01, accessed on 16 April 2025). The total hippocampal volume (V) was calculated using the following formula: V = (S_1_ + … + Sₙ) × (h + d), where S_1_ … Sₙ are the areas (mm^2^) of each slice, h is the slice thickness (mm), and d is the interslice gap (mm).

### 2.5. Behavioral Tests

Anxiety-like behavior was assessed using the open field and elevated plus maze tests. Obsessive–compulsive-like behavior was evaluated using the marble-burying test. These tests were conducted at specified time points after each stress phase. Depressive-like behavior was assessed using the Porsolt forced swim test, and for assessing hippocampal volume, magnetic resonance imaging was performed. All behavioral tests were conducted between 17:00 and 20:00, during the animals’ peak activity period, taking into account the controlled light/dark cycle of the housing conditions.

#### 2.5.1. Open Field Test (OFT)

The Open Field test was used to evaluate spontaneous locomotor and exploratory activity [14,15]. Rats were placed for 5 min in a circular white PVC arena (diameter 97 cm; wall height 42 cm; OpenScience, Moscow, Russia) under uniform illumination. Sessions were recorded and analyzed using the EthoVision XT 11.5 video tracking system (Noldus, Wageningen, Netherlands). The total distance moved (locomotor activity), number of rearings and hole explorations (exploratory activity), as well as the number and duration of freezing episodes (anxiety-like behavior) were quantified.

#### 2.5.2. Elevated Plus-Maze Test (EPM)

The Elevated Plus-Maze test is based on rodents’ natural preference for dark burrows, natural fear of being in open illuminated spaces, and fear of falling from heights and is widely used to assess the severity of anxiety in animals [16,17]. The maze consists of two closed arms and two open arms, each 50 cm long and 14 cm wide, elevated 50 cm above the floor (OpenScience, Moscow, Russia). Only the open arms were illuminated, while the closed arms were darkened. Test duration was 5 min. To assess anxiety, time spent in closed arms, freezing time, and number of head dips were calculated.

#### 2.5.3. Marble-Burying Test

The marble-burying test was used to assess digging behavior, often interpreted as a model of obsessive–compulsive behavior [18]. Nine glass marbles (diameter ~15 mm) were arranged in a 3 × 3 grid on clean bedding in one half of the test cage (“marble zone”). A rat was placed in the opposite, marble-free half, and its behavior was recorded for 10 min. The number of marbles buried (defined as being at least 2/3 covered by bedding) was counted by three independent researchers, and the average number of buried marbles per animal was used.

#### 2.5.4. Forced Swim Test (FST)

The test is based on assessing changes in animal activity in an inescapable situation and was used to assess depressive-like behavior in rats. After unsuccessful attempts to escape from the water, animals acquire a characteristic immobile posture (keeping only their head above water), which is interpreted as a manifestation of depression (“despair behavior”) [19]. Test duration was 5 min. Animals were placed in transparent plexiglass cylinders 65 cm high, 30 cm in diameter, filled 2/3 with water at 25–27 °C, and the immobility time of rats was recorded (OpenScience, Moscow, Russia).

### 2.6. Euthanasia

At the conclusion of the experiment (on day 46), animals were euthanized. To minimize suffering, euthanasia was performed under deep anesthesia via isoflurane overdose (5% in oxygen flow) followed by decapitation to ensure rapid and humane death, as well as for subsequent brain tissue collection.

### 2.7. Statistical Analysis

Data are presented as mean ± standard deviation. For statistical analysis, GraphPad Prism (version 10.0) and STATISTICA (version 12.0) programs were used, with the significance level set at *p* ≤ 0.05. For all quantitative data, the Kolmogorov–Smirnov and/or Shapiro–Wilk tests were performed to determine normality of distribution, as well as Levene’s test to determine data variance within study groups at each time point of measurements. Subsequently, for assessing intergroup differences, one-way ANOVA with appropriate post hoc tests (e.g., Tukey’s test; Dunnett’s test) was applied; if at least one of the analyses of any quantitative data describing a specific parameter yielded negative results, the Kruskal–Wallis one-way analysis of variance with subsequent Mann–Whitney rank test was applied to assess intergroup differences.

## 3. Results

### 3.1. Animal Stratification into Phenotypic Subgroups

Animal models of PTSD utilize standardized, though variable, criteria for classifying subjects to the PTSD-like group. Commonly used endpoints are changes in corticosterone levels and elevated plus maze performance; the critical window for assessing these changes falls within 1–2 weeks after exposure to a single prolonged stress protocol [5,20,21]. The inclusion criterion was based on the time spent in the closed arms of the elevated plus maze [22]. The value of this parameter in animals with PTSD should be higher than in control animals. The next inclusion criterion was the hormonal response of animals at the PTSD formation stage (single prolonged stress, Stage 1). Plasma corticosterone levels at Stage 1 should be higher than in Control group animals, indicating an acute stress response. The final inclusion criterion was the dynamics of the corticosterone level 1–2 weeks after the initial stress induction. According to the literature, animals with high anxiety show a decrease in corticosterone levels relative to the Control group during the 1–2 week period after stress exposure [6,23,24]. Consequently, the PTSD-like group comprised 20 animals that exhibited a sustained 37% increase in corticosterone levels at the first stage compared to Control group (46.43 ± 11.78 vs. 33.83 ± 10.64, *p* = 0.0017), but with a 35% decrease after 14 days (25.26 ± 11.78 vs. 38.69 ± 14.30, *p* = 0.0285). Furthermore, the PTSD-like group showed increased time spent in the closed arms of the elevated plus maze compared to the Control group (248.90 ± 26.42 vs. 199.55 ± 37.03, *p* = 0.0025). The group that experienced stress (Stress) but did not show PTSD-like behavior included 60 animals. These rats showed a 13% decrease in corticosterone levels at Stage 1 compared to the Control group (25.55 ± 11.06 vs. 33.83 ± 10.64), and by day 14 the values were comparable to the Control group (36.84 ± 17.56 vs. 38.69 ± 14.30). In the elevated plus maze test, they showed a slight decrease in time spent in the dark arms (179.48 ± 52.27 vs. 199.55 ± 37.03 *p* = 0.2068). The model success rate was 25%.

### 3.2. PTSD-like Phenotype Is Characterized by Persistent Behavioral and Physiological Changes

**Figure 1:**
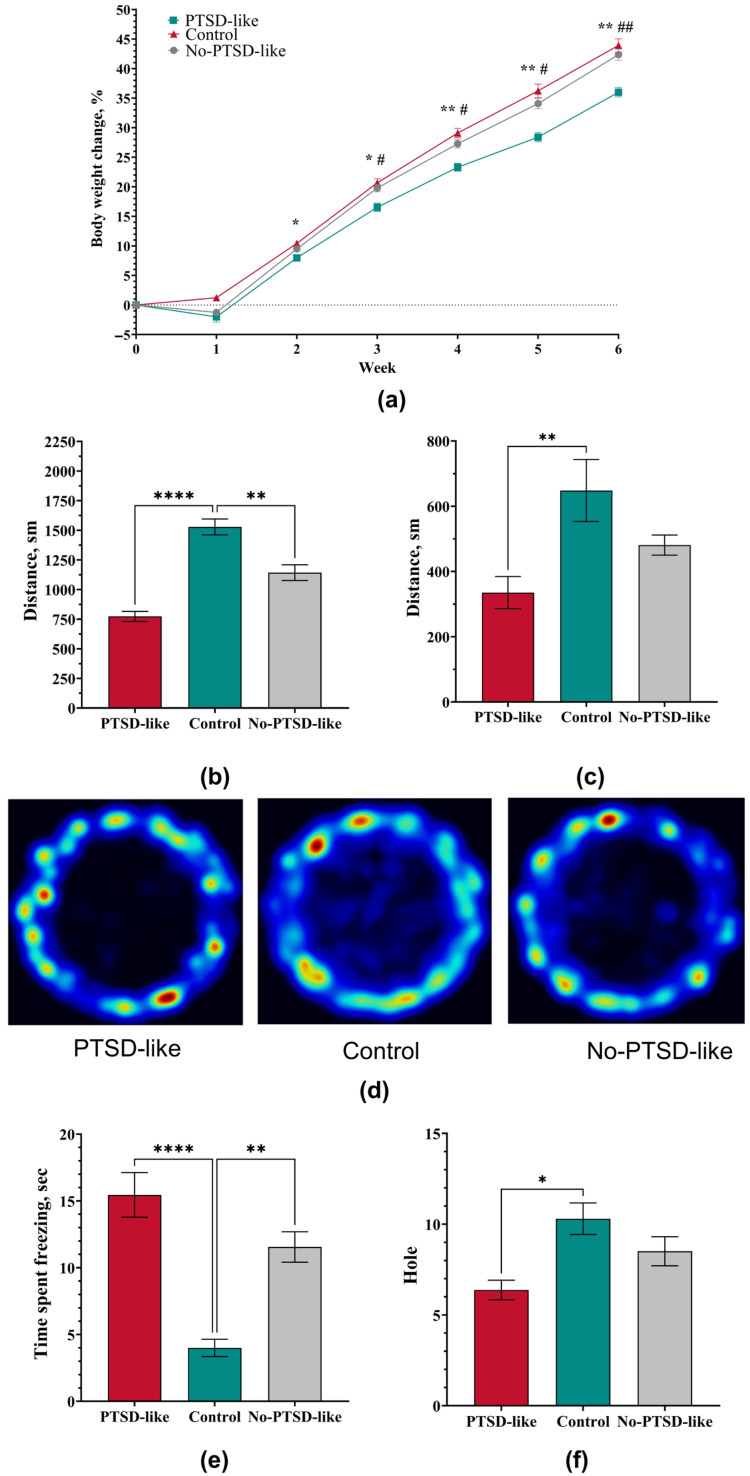
Animals with a PTSD-like phenotype demonstrated somatic and behavioral changes: (**a**) Animal body weight changes dynamics, where PTSD-like group rats showed lower weight than Control and No-PTSD-like groups. (**b**) Animals of the PTSD-like and No-PTSD-like groups showed similar behavioral impairments in the open field, characterized by a significant reduction in total distance traveled. (**c**) Animals of the PTSD-like group maintained a statistically significant reduction in total distance traveled after 4 weeks. (**d**) Graphical representation of total distance traveled by animals at the first study stage, obtained using EthoVision XT11.5 software. The red and yellow markings are the places in the open field where the animal spent the most time. (**e**) Animals of the PTSD-like and No-PTSD-like groups showed similar behavioral impairments in the open field, characterized by a significant reduction in total distance traveled. (**f**) The PTSD-like group animals showed a decreased number of head dips compared to the Control group. Data are presented as mean ± standard error of the mean. *—*p* < 0.05 compared to Control group, **—*p* < 0.01 compared to Control group, ****—*p* < 0.0001 compared to Control group, #—*p* < 0.05 compared to No-PTSD-like group, and ##—*p* < 0.01 compared to No-PTSD-like group.

Analysis of body weight dynamics revealed sustained differences among the experimental groups, reflecting the severity of stress-induced disorders (Figure 1a). During the acute stress exposure stage, all animals subjected to the SPS&S procedure showed a statistically significant decrease in body weight compared to the Control group (*p* = 0.0001, Kruskal–Wallis ANOVA by ranks). In subsequent experimental stages (reminder and extinction), all groups showed a general tendency toward weight gain. However, the rate of weight gain differed significantly among them. No-PTSD-like animals that did not develop a PTSD-like phenotype did not differ from the Control group in weight gain at stages 2 and 3 (*p* > 0.05), indicating their complete physiological compensation and restoration of metabolic homeostasis after the initial stress response. In contrast, animals with a PTSD-like phenotype demonstrated a significant reduction in weight gain throughout this study. This allows us to conclude that a persistent reduction in weight gain rate is a characteristic somatic correlate of a PTSD-like state in our model, while stress-insensitive animals are characterized by weight normalization.

In the open field test, all animals subjected to single stress exposure at Stage 1 demonstrated persistent changes in motor and exploratory activity: all stressed animals showed similar behavioral impairments characterized by a significant reduction in total distance traveled (*p* = 0.0001; Figure 1b,d) and increased freezing time (*p* = 0.0001; Figure 1e) compared to the Control. A key distinction was observed in the parameters of orienting-exploratory activity: a decreased number of head dips was observed exclusively in the PTSD-like group, where this indicator was statistically significantly lower (*p* = 0.0323, Figure 1f) than in the Control group. This indicates a more profound impairment of exploratory behavior specific to the PTSD-like phenotype.

At the 4-week time point, a dynamic shift in behavioral manifestations was observed. Animals of the PTSD-like group showed partial behavioral normalization: orienting-exploratory activity parameters and freezing time did not differ from control values. However, a statistically significant reduction in total distance traveled persisted (*p* = 0.002, Figure 1c), indicating the persistent nature of motor impairments.

Similar results are also observed in the elevated plus maze test, where behavior changes from generalized anxiety manifestations to more specific long-term consequences of stress exposure.

**Figure 2:**
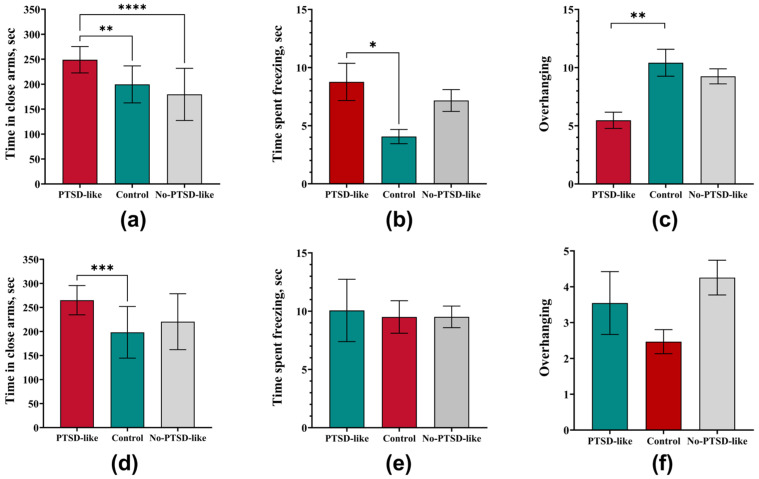
PTSD-like group animals showed high anxiety levels relative to the Control group and No-PTSD-like group. (**a**) The PTSD-like group rats spent more time in the closed arms of the elevated plus maze at Stage 1 of the experiment compared to the control group and the No-PTSD-like group. (**b**) The PTSD-like rats showed more freezing at Stage 1 of the experiment compared to the Control group and the No-PTSD-like group. (**c**) The PTSD-like rats showed fewer head dips from the elevated plus maze at Stage 1 of the experiment compared to the Control group and the No-PTSD-like. (**d**) After 4 weeks, PTSD-like rats maintained high values of time spent in the closed arms of the elevated plus maze relative to the Control group. (**e**) After 4 weeks, PTSD-like rats showed a freezing time that was not different from the Control and No-PTSD-like groups. (**f**) After 4 weeks, PTSD-like rats showed head dipping similar to the Control and No-PTSD-like groups. Data are presented as mean ± standard error of the mean. *—*p* < 0.05, **—*p* < 0.01, ***—*p* < 0.001, ****—*p* < 0.0001.

Rats with a PTSD-like phenotype demonstrated a significant increase in anxiety compared to both the Control group and animals subjected to identical stress exposure but that had maintained resistance to disorder development. The PTSD-like group showed a significant increase in time spent in the closed arms of the elevated plus maze (Figure 2a), indicating pronounced avoidance behavior. Simultaneously, an increased duration of freezing episodes was recorded (Figure 2b), as well as a significant reduction in exploratory activity assessed by the number of head dips from the open arms (Figure 2c). In contrast, animals of the No-PTSD-like group showed no significant deviations from control values in these parameters, emphasizing the specificity of the identified impairments specifically for a PTSD-like condition.

Assessment after 4 weeks confirmed the persistent nature of impairments in PTSD-like rats but revealed changes in the anxiety behavior pattern. The time spent in the closed arms remained significantly increased (Figure 2d), indicating a persistence of avoidance behavior as a key symptom. However, parameters such as freezing time and the number of head dips normalized, showing no significant difference from the Control group (Figure 2e,f). This dynamic suggests a transition from generalized anxiety to a more specific, avoidance-focused behavioral profile, which corresponds to the clinical picture of PTSD in humans [25,26,27].

At all observation stages, No-PTSD-like animals did not statistically differ from the Control group in most elevated plus maze parameters. This indicates that despite the experienced stress exposure, this group of rats possesses effective compensatory mechanisms that prevent the development of persistent PTSD-like impairments.

### 3.3. Long-Term Stress Consequences in Physiological and Hematological Markers After Exposure to the Modified SPS&S Model

Hyperglycemia is an established marker of acute stress response, pathogenetically explained by activation of the sympatho-adrenal system with subsequent secretion of catecholamines and glucocorticoids stimulating glycogenolysis and gluconeogenesis processes [28]. However, there is certain specificity in using glucose level as a criterion for a PTSD-like state within the SPS&S model. According to the protocol, the model includes usage of diethyl ether, which itself induces significant increase in blood glucose levels, thereby masking specific changes related specifically to stress response [28]. To standardize conditions for blood glucose measurement and ensure comparability across groups, all samples were collected from animals under ether anesthesia.

**Figure 3:**
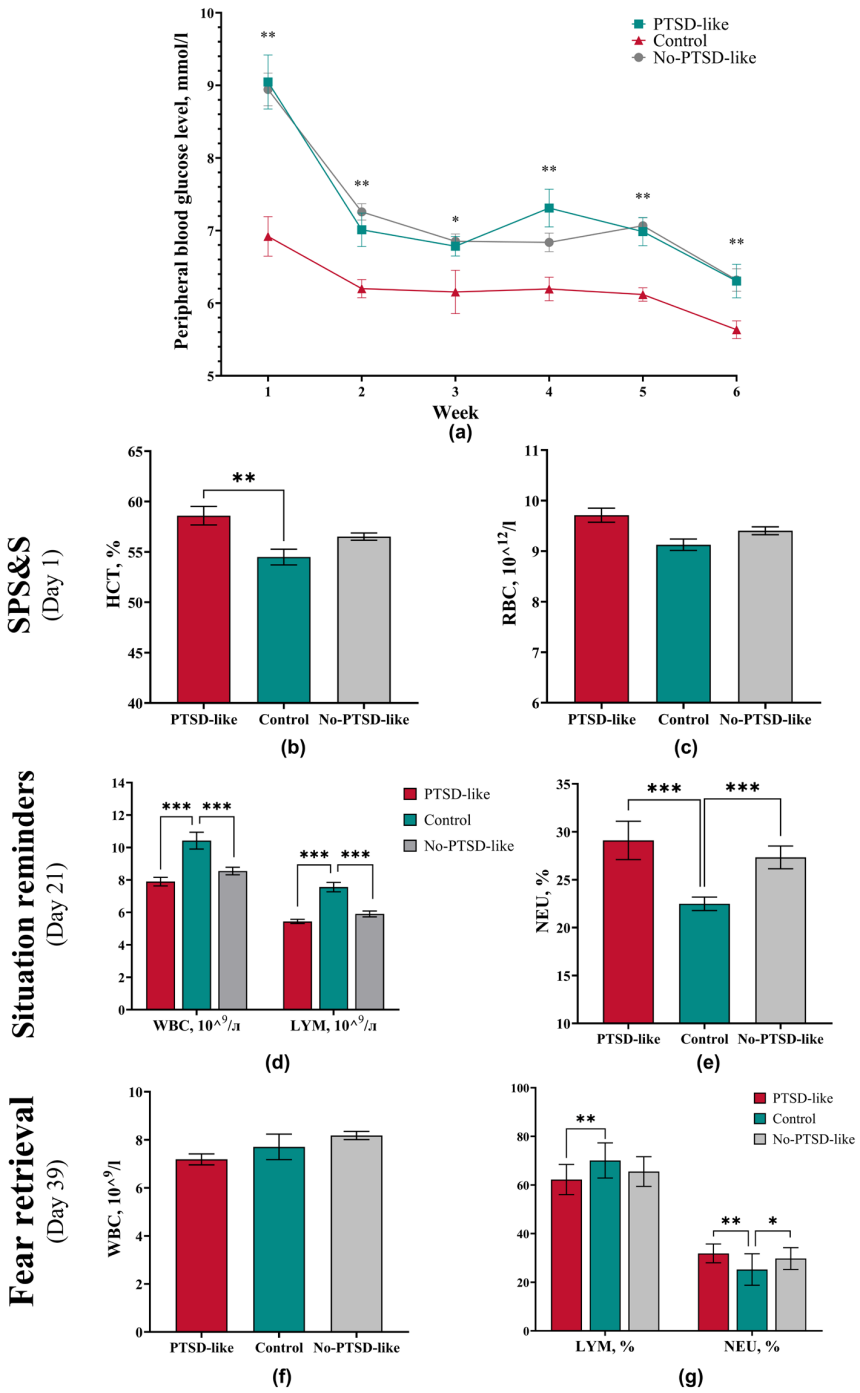
Dynamics of hematological parameters and glucose level at different stages of PTSD-like state development: (**a**) PTSD-like and No-PTSD-like animals demonstrate increased blood glucose levels relative to Control group. (**b**) PTSD-like group animals demonstrate increased hematocrit level relative to Control group. (**c**) A tendency toward increased erythrocyte count was observed (*p* = 0.06). (**d**) PTSD-like and No-PTSD-like animals demonstrate a significant decrease in total leukocyte count and absolute lymphocyte number. (**e**) PTSD-like and No-PTSD-like animals demonstrate an increased relative neutrophil level. (**f**) At the third study stage, the statistical significance of differences in total leukocyte count between groups decreased. (**g**) At the third stage, PTSD-like and No-PTSD-like animals demonstrated an increased percentage of neutrophils (*p* = 0.0289) relative to the Control group. Data are presented as mean ± standard error of the mean. *—*p* < 0.05 compared to Control group, **—*p* < 0.01 compared to Control group, and ***—*p* < 0.001 compared to Control group.

Glucose measurement results demonstrated a significant increase in blood glucose levels in animals with a developed PTSD-like phenotype and in animals without disorder signs relative to intact animals (Figure 3a). These changes persisted at all study stages, including the reminder and extinction phases.

Immediately after the single prolonged stress procedure, PTSD-like group animals showed an isolated increase in hematocrit level compared to the Control group (*p* = 0.0028) (Figure 3b). Simultaneously, a tendency toward increased erythrocyte count was observed (*p* = 0.006) (Figure 3c). As the obtained values remained within the physiological range, these findings are interpreted as stress erythrocytosis (pseudopolycythemia). This condition, characteristic of intensive physical and emotional loads, could be provoked by the combination of immobilization, forced swimming, and electrostimulation, probably through the mechanism of adreno-dependent spleen contraction and release of deposited blood into the systemic circulation [29].

Throughout the entire four-week reminder phase, PTSD-like and No-PTSD-like animals showed persistent hematological changes characteristic of prolonged stress exposure. Compared to the Control group, these animals showed the following: a significant decrease in total leukocyte count, a decrease in absolute lymphocyte number (Figure 3d), and a relative increase in neutrophil level (Figure 3e). This picture of neutrophilic leukocytosis with lymphopenia is a classical marker of glucocorticoid exposure and was reliably reproduced throughout all four weeks, indicating the persistent nature of the stress response [30,31,32,33].

At the extinction stage, the intensity of observed changes decreased. The statistical significance of differences in total leukocyte count between groups diminished (Figure 3f). However, PTSD-like group animals maintained a statistically significant decreased percentage of lymphocytes (*p* = 0.0023) and an increased percentage of neutrophils (*p* = 0.0013) relative to the Control group (Figure 3g), indicating residual activity of stress-realizing systems even after cessation of direct stress exposures. The No-PTSD-like showed an increase only in relative neutrophil level (*p* = 0.0228) (Figure 3g).

Analysis of plasma ACTH and corticosterone concentrations revealed complex and divergent dynamics of hypothalamic–pituitary–adrenal (HPA) axis activity in animals with different responses to stress exposure. For analysis, not absolute but relative values were used, where the mean value of the Control group at each week was taken as 1. This is necessary to correct for circadian rhythm-related fluctuations in ACTH and corticosterone levels in the dynamics.

**Figure 4:**
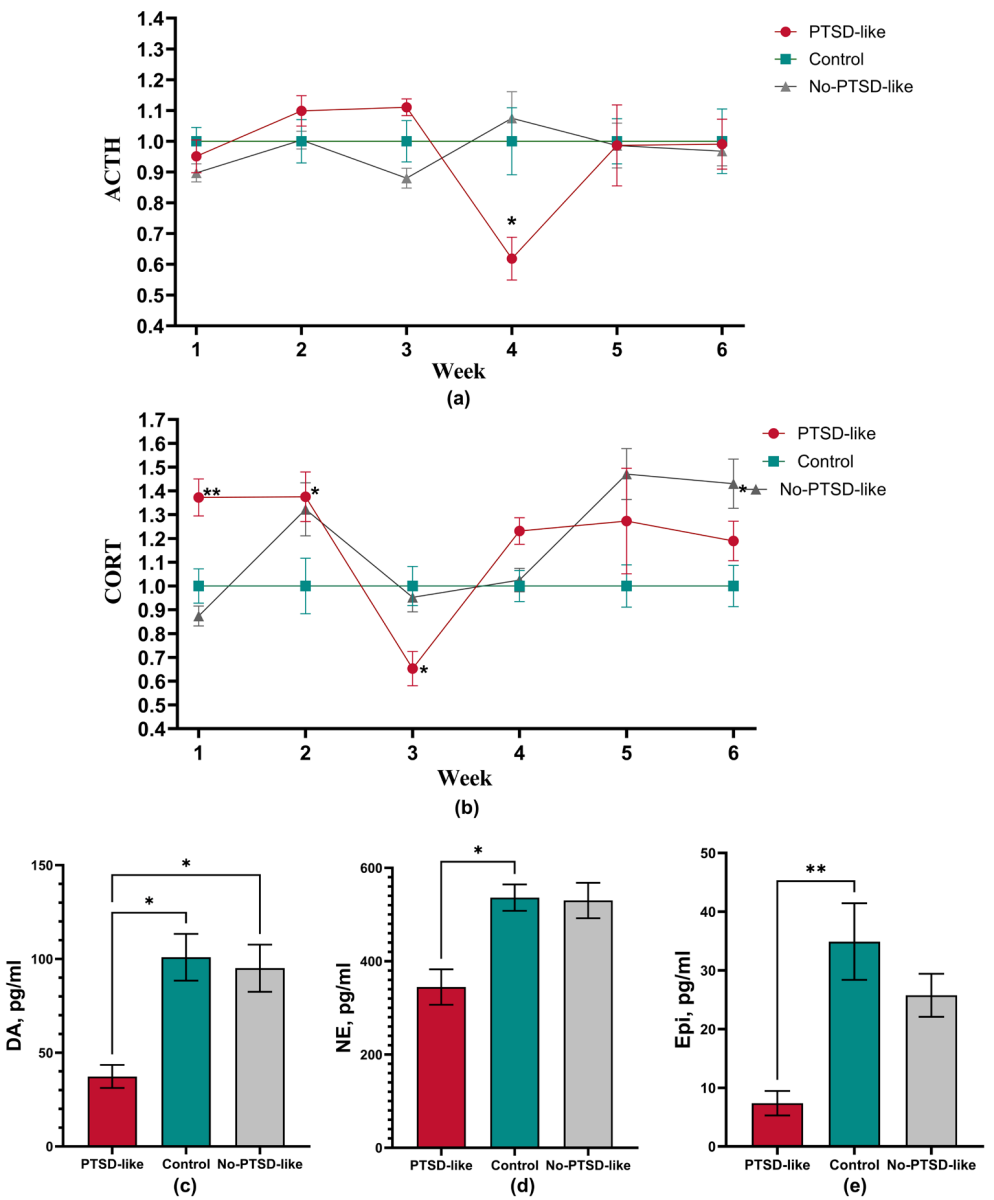
Results of enzyme-linked immunosorbent assays (ELISAs): (**a**) ACTH level in blood plasma. PTSD-like group animals demonstrate a 35% decrease in ACTH level at week 4 of this study compared to the Control group. No-PTSD-like animals demonstrated stable ACTH levels. (**b**) Corticosterone level in blood plasma. PTSD-like group animals demonstrated an increased corticosterone level at weeks 1–2, a significant decrease in CORT level at week 3, and an increase by the end of this study. No-PTSD-like animals demonstrated a delayed stress response in the form of an increased corticosterone level at week 2, normalization of corticosterone values at weeks 3–4, and a significant rise by week 6. (**c**) PTSD-like group animals demonstrated a decreased dopamine level relative to the Control group. (**d**) PTSD-like group animals demonstrated a decreased norepinephrine level relative to the Control group. (**e**) PTSD-like group animals demonstrated a decreased epinephrine level relative to the Control group. Data are presented as mean ± standard error of the mean. *—*p* < 0.05 compared to Control group, **—*p* < 0.01 compared to Control group.

Analysis of HPA axis hormone dynamics revealed a complex, three-stage pattern of changes (Figure 4a). By weeks 2–3, an initial increase in ACTH level by 10% above baseline is observed. Of particular interest are changes by week 4 of this study, namely a sharp decrease in ACTH level by 35% compared to the Control group (*p* = 0.0485), coinciding with the reminder phase. At weeks 5–6, ACTH level normalized to control values. In the acute phase, the PTSD-like group demonstrated an increased corticosterone level by ~37% (*p* = 0.0017, Figure 4b). Of particular importance is the corticosterone level at week 3, where the PTSD-like group demonstrated a 35% decrease in hormone level (*p* = 0.0285). By weeks 5–6, an inversion of the hormonal profile was observed, with corticosterone level exceeding control values by 20–25%. It should be noted that in the acute phase, changes included the expected increase in corticosterone level correlating with the initial ACTH rise. But at week 3, a clear HPA axis imbalance is observed: against a background of still elevated ACTH, corticosterone level unexpectedly decreased. This could indicate the beginning of adrenal cortex insufficiency, but this condition does not typically develop so quickly. Therefore, this instead indicates a change in adrenal cortex sensitivity with an alteration of negative feedback mechanisms, as evidenced by week 4 data. Results of weeks 5–6 also refute the possibility of adrenal insufficiency. The elevated corticosterone levels observed at the end of this study instead indicate relative autonomy of the peripheral HPA axis link (regulation by “extrapituitary” mechanisms—potentially involving inflammatory mediators, which were not assessed here) or that adrenals have acquired increased sensitivity to normal ACTH levels. It should also be noted that postmortem analysis revealed the PTSD-like group had decreased levels of dopamine (*p* = 0.0276, Figure 4c), norepinephrine (*p* = 0.0117, Figure 4d), and epinephrine (*p* = 0.0017, Figure 4e) compared to the Control group. This indicates an impairment of catecholamine synthesis processes, likely mediated by corticosterone’s inhibitory action on enzymes involved in their biotransformation.

No-PTSD-like animals demonstrated relatively stable ACTH levels, close to control values with minor fluctuations (Figure 4a), indicating preservation of mechanisms for the basic regulation of pituitary function in these animals. In the acute stress phase (Week 1), the No-PTSD-like showed a 13% decrease in corticosterone level compared to the Control group (Figure 4b). Animals of this group exhibited a delayed stress response, as by week 2 a tendency toward a 30% increase in corticosterone level was noted, which explains the leukocyte changes observed in the peripheral blood of animals. At weeks 3–4, the No-PTSD-like showed values close to control. By the end of this study, animals that were insensitive to PTSD-like state formation showed, in contrast to the PTSD-like group, a 45% increase in corticosterone level (*p* = 0.0320). For this group, the key change is high adrenal activity at normal ACTH values, which also points to organ autonomy in the long-term after stress induction. Interestingly, unlike the PTSD-like group, No-PTSD-like animals showed no significant changes in catecholamine levels in the hippocampus (Figure 4c–e), which may reflect preservation of neurochemical balance in key brain structures in stress-resistant individuals.

### 3.4. The Modified SPS&S Model Reproduces an Isolated PTSD-like State Not Accompanied by Comorbid Depressive or Obsessive–Compulsive Manifestations

**Figure 5:**
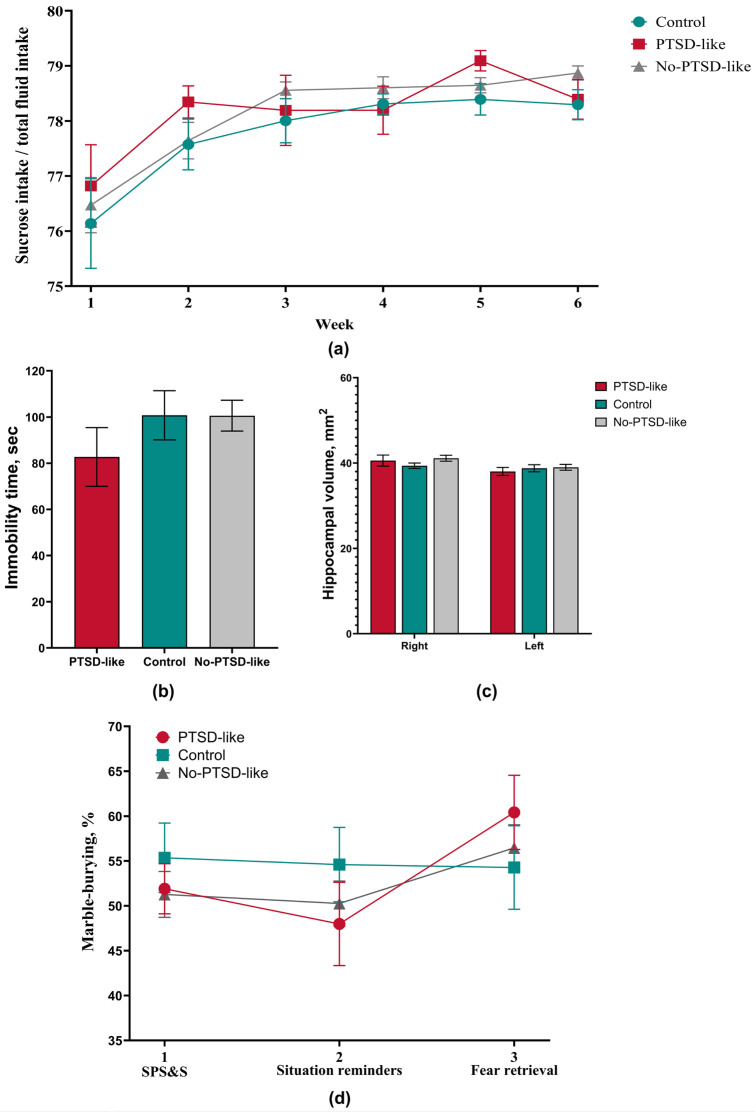
The modified SPS&S model is not accompanied by depressive and obsessive–compulsive manifestations: (**a**) PTSD-like and No-PTSD-like animals did not demonstrate changes in sucrose consumption compared to the Control group. (**b**) PTSD-like and No-PTSD-like animals did not demonstrate changes in immobility time in the forced swimming test compared to the Control group. (**c**) PTSD-like and No-PTSD-like animals did not demonstrate changes in hippocampus volume compared to the Control group. (**d**) PTSD-like and No-PTSD-like animals did not demonstrate changes in the marble burying test compared to the Control group at each study stage. Data are presented as mean ± standard error of the mean (SEM).

To assess the presence of anhedonia as a key symptom of depression-like state, the sucrose preference test was used. The results revealed no statistically significant differences between the experimental groups. Notably, throughout the entire experiment, sucrose solution consumption in PTSD-like and No-PTSD-like animals exceeded that of the Control group (Figure 5a). The absence of a decrease in sucrose consumption, as well as its excess in experimental groups, allows us to exclude anhedonia as a component of the behavioral phenotype in this model.

To assess the presence of depression-like behavior characterized by the “behavioral despair” phenomenon, the forced swimming test was applied. Statistical analysis did not reveal a significant increase in immobility time in experimental group animals compared to the Control group (Figure 5b). According to classical interpretation, this result indicates the absence of depression-like behavior. However, interpretation of test results in the context of this work requires consideration of methodological features of the model used. The SPS&S protocol includes a 20 min swimming stage, which may influence the behavioral response of animals during subsequent testing in this apparatus. This result is an additional, but not key, argument in the assessment of the induced PTSD-like phenotype.

This confirms that within the modified SPS&S model, the stress exposure primarily leads to functional, rather than structural, impairments. Since a decrease in hippocampus volume represents a stable neurobiological correlate of depressive disorders, morphometric analysis of MRI images was performed to assess structural changes in this area. Statistical analysis did not reveal significant differences in hippocampus volume between the animal group with a PTSD-like phenotype and the Control group (Figure 5c). This confirms that within the modified SPS&S model, stress exposure leads predominantly to functional rather than structural impairments.

To identify obsessive–compulsive symptomatology characterized by stereotypical repetitive actions, the marble burying test was used. Data analysis did not reveal statistically significant differences in the number of buried marbles between stressed animal groups and the Control group (Figure 5d). Based on the obtained data, a compulsive component can be excluded from the structure of the developed behavioral disorder. The absence of increased compulsive activity is consistent with clinical observations, according to which obsessive–compulsive symptomatology is not an obligatory component of post-traumatic stress disorder and develops only as a comorbid condition under certain circumstances [9].

## 4. Discussion

This study presents the results of modeling post-traumatic stress disorder through a targeted modification of the classical Single Prolonged Stress (SPS) model and the implementation of phenotypic stratification. This approach not only replicated key PTSD symptoms but also captured the clinically relevant heterogeneity of the trauma response. Although considered a gold standard, the classical SPS model is often criticized for the transient nature (short duration and spontaneous resolution) of its behavioral and hormonal changes—a limitation that restricts its utility for modeling chronic PTSD [6,34]. This model accurately captures the acute stress disorder state observed in the initial weeks post-trauma, which is characterized by pronounced autonomic and behavioral reactions—as we observed in all animals at the first stage. However, within the classical protocol, these acute reactions often diminish over time without progressing into a persistent pathological state

A key improvement in our study was the introduction of reminder and extinction phases, which effectively addressed this issue of transience. The classical model answers the question “What happens immediately after trauma?”, while our modification addresses a central question of clinical psychiatry: why does an acute condition progress into chronic PTSD in some patients, while resolving in others? In this study, the reminder phase specifically models this critical transition, wherein a patient attempting to function in society experiences constant triggering of pathological trauma memories, which impedes the spontaneous extinction of the stress response and leads to the development of persistent avoidance symptoms [35]. Furthermore, the “extinction” phase in our model essentially represents an analogue of the development of post-traumatic personality changes, whereby maladaptive mechanisms lead to social alienation, corresponding in clinical practice to the most resistant and severe forms of the disorder [36].

The stratification criteria based on corticosterone dynamics and behavior in the elevated plus maze (EPM) have proven highly effective in many PTSD models, and our model is no exception [22]. Importantly, and in contrast to the transient effects typically observed in the classical SPS model, the pathological changes in our modified SPS&S protocol were persistent. The PTSD-like group demonstrated the classical PTSD dynamics of the HPA axis: an excessive response to acute trauma with subsequent development of hyporeactivity (evidenced by decreased corticosterone at week 3), and this hyporeactivity persisted throughout the observation period, indicating a successful mitigation of the transient nature typically associated with endocrine alterations in the standard model. In humans, these changes clinically manifest as the rapid consolidation of the core PTSD pathology—in these patients, the anxiety stage with high cortisol is almost immediately replaced by a stage of exhaustion with characteristic HPA axis hypofunction, which correlates with their avoidance behavior [37]. This condition is described in PTSD patients as an “exhaustion phenomenon” of the HPA axis [38]. In contrast, the No-PTSD-like, despite the experienced trauma, retained the ability to adapt and demonstrated a delayed but more physiological hormonal response. However, their resilience has limits, as evidenced by the increase in corticosterone level by week 6. In clinical practice, a similar condition is observed in people who, months or even years after trauma, while continuing to live in a state of chronic stress [39,40] (e.g., persistent financial difficulties; social isolation), begin to demonstrate symptoms like burnout syndrome. Such patients develop hypercortisolemia, leading to metabolic disorders, anxiety, and irritability. Thus, the model allows for the assessment of two fundamentally different conditions: rapid PTSD formation in vulnerable individuals and a slow “resource depletion” with the risk of developing somatoform and depressive disorders in initially resistant individuals. This explains the diversity of diagnoses observed in populations of trauma survivors.

The persistent neutrophilic leukocytosis with lymphopenia found in “vulnerable” rats is a likely consequence of prolonged elevation of endogenous glucocorticoid levels [41]. Corticosterone reduces the expression of cellular adhesion receptors and the synthesis of chemoattractants on leukocytes and also inhibits neutrophil apoptosis while promoting lymphocyte apoptosis [42,43]. Additionally, corticosterone can inhibit the organic cation transporter 3 (OCT3), which normally facilitates the transport of norepinephrine, epinephrine, and dopamine, thereby altering their bioavailability in the brain [44]. Consistent with elevated corticosterone levels, the PTSD-like group showed a significant decrease in the levels of mediators of the dopamine → norepinephrine → epinephrine cascade. Importantly, stress-resilient animals maintained monoamine balance. A decreased norepinephrine level in the hippocampus in patients can lead to concentration difficulties, ADHD-like symptoms, memory impairments, loss of motivation, and feelings of indifference [45,46]. It is plausible that the reduction in these hippocampal monoamines represents a protective mechanism against traumatic memory retrieval. Many researchers report alterations in hippocampus volume associated with PTSD [47], but in our study, the PTSD-like group showed no significant changes in hippocampus volume. This indicates that in the early stages of the disorder, functional deficits rather than structural impairments predominate. This is consistent with data from some MRI studies in patients with recently diagnosed PTSD (without significant comorbid conditions) [48,49] and emphasizes the potential reversibility of these processes with timely intervention (such as psychopharmacotherapy or cognitive-behavioral therapy).

Thus, the most important feature of this modified protocol is its ability to overcome the transience characteristic of the classical SPS model. The long-term preservation of behavioral, hematological, and endocrine alterations characteristic of the PTSD-like phenotype for 6 weeks offers a novel and valuable platform for more profound and extended investigation of new psychotropic drugs and the study of the underlying pathophysiological mechanisms of the disorder. Moreover, the reminder phase not only prolonged and stabilized the PTSD-like phenotype but also, interestingly, enhanced its selectivity by minimizing comorbid depressive and OCD-like manifestations.

An important limitation of the present study is the use of only male rats. Considering the known sex differences in the epidemiology, symptomatology, and neurobiology of PTSD, as well as the modulating influence of gonadal hormones on HPA axis response and immune function, our results cannot be directly extrapolated to females. The use of a single-sex cohort at this stage was justified by the task of primary validation of the modified model and minimizing variability associated with the estrous cycle. However, identifying potential sex differences in the development of PTSD-like phenotype, biomarker dynamics, and therapeutic intervention efficacy within the proposed model represents a crucial direction for future research.

We acknowledge the limitation of the present study regarding the use of only male rats and have added this to our manuscript: “Considering the known sex differences in the epidemiology, symptomatology, and neurobiology of PTSD, as well as the modulating influence of gonadal hormones on HPA axis response and immune function, our results cannot be directly extrapolated to females. The use of a single-sex cohort at this stage was justified by the task of primary validation of the modified model and minimizing variability associated with the estrous cycle. However, identifying potential sex differences in the development of PTSD-like phenotype, biomarker dynamics, and therapeutic intervention efficacy within the proposed model represents a crucial direction for future research.”

## 5. Conclusions

The modified SPS&S model with reminder and extinction phases represents a valid and highly specific tool for modeling a long-term PTSD-like phenotype without comorbid depressive and obsessive–compulsive symptomatology. Phenotypic stratification into PTSD-like and stress-resilient individuals effectively recapitulates the clinical heterogeneity of PTSD and allows for the investigation of biological underpinnings of individual stress susceptibility. This model is promising for the preclinical evaluation of new therapeutic strategies aimed at the treatment of post-traumatic stress disorder.

## Data Availability

The original contributions presented in this study are included in the Supplementary Material. Further inquiries can be directed to the corresponding author.

## Supplementary Materials

The following supporting information can be downloaded at: https://www.mdpi.com/article/10.3390/biomedicines14020356/s1. File S1: Detailed Protocol for Sublingual Vein Blood Collection in Rats.

## Abbreviations

The following abbreviations are used in this manuscript:

ACTH	Adrenocorticotropic hormone
ADHD	Attention deficit hyperactivity disorder
ELISA	Enzyme-linked immunosorbent assay
EPM	Elevated plus-maze test
FST	Forced swim test
HPA axis	Hypothalamic–pituitary–adrenal axis
MRI	Magnetic resonance imaging
OCD	Obsessive–compulsive disorder
OCT3	Organic cation transporter 3
OFT	Open Field test
PTSD	Post-traumatic stress disorder
SPS&S	Single Prolonged Stress with Subsequent Stress
SPT	Sucrose preference test
