# Supplementary material for: From Acute Stress to Long-Term Dysregulation: Changes in Hematological and Hormonal Parameters in the Long-Term Post-Stress Period in a Modified SPS&S Model
Source: Biomedicines. 2026 Feb 3;14(2):356. doi: 10.3390/biomedicines14020356 (PMC12938505; doi:10.3390/biomedicines14020356)
Supplement: Supplementary file 1 [file biomedicines-14-00356-s001.zip › biomedicines-4044985-supplementary.pdf]

Detailed Protocol for Sublingual Vein Blood Collection in Rats

Blood Collection Procedure

Blood collection from animals was performed with measures to prevent the onset and development of side effects such as hypovolemic shock and anemia. Blood was collected weekly (not exceeding 7.5% of total blood volume), considering that blood composition recovery occurs according to the formula: 1 ml/kg/day.

Sublingual Vein Blood Collection Technique

The procedure was performed as follows:

1. Animal Anesthesia. The procedure was performed on an anesthetized animal. The animal was anesthetized with inhalational isoflurane anesthesia (5% in oxygen flow).

2. Tongue Extraction. The tongue was carefully extracted from the mouth of the anesthetized animal by rotating a cotton swab.

3. Vein Visualization and Puncture. Veins were visualized and a puncture was made with a scarifier using a sharp movement (across the vein).

4. Blood Collection. The animal was held by the scruff, with the skin gathered and slight pressure applied to the neck vessels, then turned head down over the tube. The first drop was left on a napkin to prevent it from entering the sample. Blood was then collected

5. Post-Collection Procedure

The scruff was released to reduce venous return, and the rat was placed on its side on a napkin to prevent blood from the oral cavity from flowing into the lungs and esophagus. A cotton tampon was applied for sufficient time to stop bleeding.

Sample Processing.

Immediate Processing.

One drop of blood was immediately used for glucometry 20 µl was retained for hematological analysis

Centrifugation. The remaining blood was centrifuged for 5 minutes at 3000 rpm.

Serum Preparation. The obtained serum was aliquoted into 55 µl portions in tubes for enzyme-linked immunosorbent assay (ELISA).

Sample Dilution

If there was insufficient material for ELISA analysis, it was diluted with physiological saline.
